# Richmond and Louisville Medical Journal

**Published:** 1872-04

**Authors:** 


### THE RICHMOND AND LOUISVILLE MEDICAL JOURNAL.

> " Now, in the name of all the gods at once,
> Upon what meat doth this, our Ceasar feed,
> That he is grown so great?"

The Medical Chesterfield of the American Medical Press—the oracle of everything professional—who sits upon the editorial tripod of the *Richmond and Louisville Medical Journal*, has had his nerves most terribly shocked, and his *toes* most painfully crushed, by the (supposed) *youthful* indiscretions of The Companion, and his wailings and laments fill the air. We cry to the "hills and rocks" to "hide" us from his wrath! Who is able to "abide" it?

It is true, The Companion is only a little over twelve months old. It has not the age, the herculian grasp of intellect or the wrathful energy, doubtless, of the "largest Medical monthly in America," yet, while *deploring its deficiencies* in these and other respects, it has never deliberately, wantonly and without provocation, assaulted and assailed any Medical man or journal. On the contrary—as our exchanges and readers will bear witness—it has uniformly spoken in terms of gentlemanly and professional courtesy of *every journal*, and especially of the *Richmond and Louisville Medical Journal* and its editor. No word of censure or ungenerous reproach has yet marked its pages. It has never yet (nor ever will) deemed it necessary to secure success by resorting to detractions of, and unkind flings at other journals. It does not desire pecuniary benefits, or any other, at the sacrifice of that manly and professional dignity, which *fails* to take pleasure in the success of a brother in the same field of labor. This spirit it has shown on all occasions—and yet, notwithstanding this uniform courtesy, and engaged in attending strictly to its own affairs, it has called up the ire, and crushed the toes of the "largest Medical Monthly in America." Woe unto us! that the Chesterfield of the medical press had toes at all—since these appendages, crowned with hypertrophied cuticle, have been so unmercifully and so innocently injured by us.

"The largest Medical Monthly in America" avers that THE COMPANION has been "*prating*," (that's the word) of ethics. Has it? Does our Solon, with immaculate tone, call it "*prating*" to reverence and defend the Code of Ethics, and to advise obedience to its precepts? And—bless its code-loving soul—has THE COMPANION nauseated its delicate stomach—the stomach of "the largest Medical Monthly in America," the editor of which, for some years back, has ridden the noble (now ignoble, our *prater* thinks) beast to his own ineffable delight? Does the stand-point from which "the largest Medical Monthly in America" (*if not* the "*cheapest* and the *best*") estimate "*prating*" reveal the *real value* and *true colors* of the sublime ethical radiations of its irreproachable and peerless tripod! "You know how it is yourself," most wonderful and voluminous of "*praters;*" and however sure of ethical infallibility you may deem yourself—yet, not by our consent will we allow *your measure of yourself* to be used as the *true standard for us*—even were you, which you seem to think, the Medical Pope, himself?

"Self-laudation and boasting," says the Medical Chesterfield of "the largest Medical Monthly in America" is—"disreputable!" Mark it! it is disreputable to "boast"—the paragon of Medical journalism being *the judge;* and yet, "tell it not in Gath, publish it not in the streets of Askelon"—on every cover of the *Richmond and Louisville Medical Journal*, is endorsed and proclaimed in large type—"THE LARGEST MEDICAL MONTHLY IN AMERICA!"

Now, that's not "boasting," not a bit of it—it's the biggest sort of bragging, only! It is worse. It may be true that it is "the largest Medical Monthly in America," but is it *fair*, is it *just*, (our accuser being the judge), to make the impression, so continuously urged, that *no other journal* in America publishes as much Medical matter *Monthly?* The Medical weeklies, and semimonthlies are thus injured (our Chesterfield so asserting) by "the largest Medical Monthly in America" which, in contrast with them, is not the "*largest*," "*cheapest*" or "the *best*."

"Boasting" is "disreputable," is it? What *does our Chesterfield* say *in* and *about* "the largest Medical Monthly in America?" Listen: "Nearly two hundred subscribers have been added to the subscription list since the closing weeks of the old year and the opening weeks (ah! it seems tautology is *more tolerable* than typographical errors!) of the new. Each mail brings some additions. The January edition has been entirely exhausted. * * * * Earnest thanks are returned for this welcome co-operation in the effort to establish a *large* (italics ours) Medical journal in the Southern States." Again:

"Many canvassers of this journal ("the largest Medical Monthly in America") are so active in its support that they are giving their commissions to new subscribers, (What! do we hear aright? and *this is not boasting!!*) as an inducement in securing their influence and assistance. * * * * No one will, after reading these facts, be surprised (however much they may be at the bragging), to learn that the subscription list has recently had an accession of over two hundred names, and the January, February, and almost all (oh! *how* our Chesterfield *does "pile it on!!"*) of the March numbers have been exhausted,'—*and his "boasting" to boot!*

We would have even the editor of "the largest Medical Monthly in America" to know that we do not think it our duty to tyrannize over his conscience. We have not a word to utter in condemnation of all this, *it is his business, not ours*, and we have been measuring "our Chesterfield" simply by his own measuring-stick. If he *cannot wear* the garments *he cut* for himself, it is certainly exceedingly "bad taste" and a woeful want of "decorum, ethics or propriety," to require others to do it for him.

Moreover, THE COMPANION has committed "a breach of good taste" in publishing "commendatory letters." We prefer having a better, a more consistent teacher of "taste" for our guidance, and besides, we have never applied to or besought, the self-appointed Chesterfield of the Medical Press, to teach us his manners. In fact, he is not our criterion, and never can be.

We are rejoiced that our efforts to serve the profession have received their approval. We have not labored for pecuniary reward, and were the subscription list of THE COMPANION "the largest in America," it would not add one dollar to our pocket. THE COMPANION is the journal of the profession. It was established *for their benefit*. They have (and shall continue to have) as much right to it, as we. Its pages belong to all—are open to all, if honorable and true—who may desire to reach their brethren through it, and if our friends desire to communicate the fact of *their* appreciation of it to one another, we shall not debar them the privilege, even at the command of "the largest Medical Monthly in America." It is *above* and *beyond* the supercilious adjudgments of would-be Chesterfields or Medical Martinets.

But, says our "*prating*" accuser, THE COMPANION has "violated *decorum, good taste, ethics and propriety!!*" Æsop tells us of a fatherly crab, who, with sanctimonious air, and sapient look, addressed his brood of young crabs, as follows: "My children, I am *astonished* and *mortified* at the *oblique* manner of your walking. I beg you *go straight-forward.*" One of the *youthful* crabs, not having the fear of his crab-ish lordship before him, remarked—"Ah! but noble sire, *why don't you* give us *an example* to *back your precept?* You who walk *obliquely, do you ask us to walk straight?*" A preacher of "good taste" etc., would do well to put himself *fully up* to the mark of propriety before venturing to lecture others. Who is it then that, without cause or provocation, holds up to Medical ridicule, three Medical journals, and the State Medical Society, of Virginia,* in

---

* NOTE.—In the notice of the editor, of the Society referred to, he seeks to put a construction on the course of this journal, as wide of the truth as it was one number of his journal? Suppose the *Galveston Medical Journal* does hybernate, and the Secretaries of the Virginia State Society write in all the persons known to grammar—shall this teacher of "decorum and good taste" blaze it forth to the world? Was "good taste," "ethics or propriety" displayed when failing to defeat a townsman as President of the American Medical Association, he suddenly takes a paroxysm of "ethical" fervor—save the mark—and denounces him through the press? Was Yandell less guilty—if guilty at all—*after* than *before* his election? What right has this "Pope" over his brethren, by virtue of which his decrees shall have the value of *law?* If Dr. Yandell deserved professional censure, it does seem that the State Society of Kentucky might have relieved even the editor of "the largest Medical Monthly in America." of that duty—or perhaps that Society, in view of the lofty genius of the man—one of that class who believe themselves endowed with that marvelous infallibility to which Arch-Angels do not aspire, whose mental decrees leap with unerring accuracy without the necessity of an appeal to the *foolishness* of logic, to the truth of every proposition—had delegated that *trifling* duty to him!

Was it "good taste" to censure the Medical College of Mobile for opening its doors free to students, when *he*, as Dean of a Medical College "advertises" —yes, advertises—"using" as he writes, "the very means made disreputable by every nostrum-vender in the land"—the largest beneficiary list of any other College in the Union! Would it be believed that scholarships are sold by him? Would it be believed, when anathamatizing his brethren of Mobile, he publishes that "one beneficiary student" will be received from each Senatorial district of Kentucky; one from each Congressional district of the different States," and all the sons of deceased physicians? Suppose his College should become, as advertised, the Mecca of all the "beneficiaries" invited, how many paying students could his institution accommodate? Not one! and yet, Mobile must do otherwise—or be gored by "the bull" of the pope.

But it is "bad taste," etc., to publish "commendatory letters." That "squelches" us, and a few other Medical journals besides. But

> "While the lamp holds out to burn,
> The vilest sinner may return,"

and therefore, we hasten to place our literary and political editors on their guard—there is a veritable Chesterfield after them, a full fledged "pope" infallible in everything concerning the proprieties of life and journalism.

Gentlemen—we beseech you, yea, implore you, one and all, from this date

---

uncalled for. No word uttered by us was ever intened to reflect upon our Virginia friends. On the contrary, on every occasion we have, truly and conscientiously, given them and their noble Society our sincerest approval and endorsement. The object of our rampant critic is transparent. We do not and have not esteemed the Virginia Medical Society, or its members less, because of its action in reference to THE COMPANION; and on no occasion have we even alluded to it or its members "as fallen." The mistake our critic (and theirs) makes, is this—he wants to "put" us "in his shoes." What he would have done under similar circumstances, he thinks others would do. Again, he has gaged us wrong by measuring us by himself—that's all. The gentlemen of Virginia appreciated our motives and we fully appreciated their action. Our action was only a matter of courtesy which was thoroughly understood.

to cease the publication of "commendatory letters." Fie on you! you should do better. In future, before venturing upon the custom and habit of the press, do, we urge you, consult the oracle at Louisville—and should you take his advice, by all means do not copy his inconsistencies. Having cautioned our literary and political friends of the press, we turn to our medical friends, and especially our friend Dr. Butler, of the *Medical and Surgical Reporter*. We beg him to take warning. It is true we are "just a little over one year old," and the *Reporter* has seen its twenty-fifth volume, and has, year after year, published "commendatory letters," still we warn him—he may yet offend the Pope and be—anathamatized. But the offence of THE COMPANION "is rank," it "smells to heaven," and what was tolerated in the *Reporter* for years, is the most heinous of sins in THE COMPANION. Does he love us more and the *Reporter* less to have tolerated the thing in all others, *except in our case*, for years?

But this is not all. It is a mortal †offence that THE COMPANION "has reached the vigerous life of sixty-four pages and has already sixty-eight editors." The ' pope" declares it an "imposition" on the profession to tolerate—so many editors. Is it an imposition? Does the "medical press protest against it? "The largest Medical Monthly in America" so asserts: *No other* Medical journal has protested against it, *ergo*, "the largest Medical Monthly in America" is "THE MEDICAL PRESS of THE COUNTRY!!" We entreat our brethren of other medical journals to hide their diminutive heads while the eagle of American Medical journalism soars aloft—and overshadows the continent,

Or, to change the figure:

* * * "He doth bestride the narrow world,
Like a Collossus; and we, petty men
Walk under his huge legs!"

"The medical press" *does* protest against it"—that is *law;* and therefore, under such a "protest," the profession will expect the infallible law-maker to at once decapitate every fraction of his editors, and hang his name alone on the cover of his journal, "for they seem never to work." The medical "pope" *evidently* not "aparently" "tyrannizes over the fractional"—the 'fractional' rarely if ever, putting in an appearance, having gone to "rest," leaving the faultfinding, snapping, growling "head" alone in his glory.

And then THE COMPANION has "one" typographical error. "The largest Medical Monthly in America" is faultless—ah! it is a pity, since it exults so much over an "h," that it failed to remember Yandell's thrust—that if, master of every other accomplishment, our Chesterfield "could not write English!"

THE COMPANION places itself upon the ethics. By this it will be judged. It does not desire discussion, save that by it the interests of the profession may be advanced. If, therefore, the editor of "the largest Medical Monthly in America" desires, for the good of the profession, to discuss ethical questions, we accept the "gage of battle" and enter the "lists." "To the law and the testimony."

---

† The supposition here is that THE COMPANION will always be a sixty-four page journal. We shall see.

